# A Novel Finding of Sentinel Lymphatic Channels in Early Stage Breast Cancer Patients: Which May Influence Detection Rate and False-Negative Rate of Sentinel Lymph Node Biopsy

**DOI:** 10.1371/journal.pone.0051226

**Published:** 2012-12-04

**Authors:** Minghai Wang, Wenbin Zhou, Yingchun Zhao, Tiansong Xia, Xiaoming Zha, Qiang Ding, Xiaoan Liu, Yi Zhao, Lijun Ling, Lin Chen, Shui Wang

**Affiliations:** 1 Department of Breast Surgery, The First Affiliated Hospital with Nanjing Medical University, Nanjing, Jiangsu, China; 2 Department of General Surgery, The First Affiliated Yijishan Hospital with Wannan Medical College, Wuhu, Anhui, China; University of Porto, Portugal

## Abstract

**Background:**

The exact lymphatic drainage pattern of the breast hasn't been explained clearly. The aim of this study was to investigate the sentinel lymphatic channels (SLCs) in the cancerous breast. Whether the type of SLCs influenced the detection rate and false-negative rate of SLNB was also assessed.

**Methodology and Principal Findings:**

Mimic SLNB was performed in 110 early-stage breast cancer patients with subareolar injection of blue methylene dye intraoperatively. Postoperatively, 110 specimens of modified radical mastectomy were examined for all blue SLCs after additional injection of methylene dye in peritumoral parenchyma. Interestingly, three types of SLCs, including superficial sentinel lymphatic channel (SSLC), deep sentinel lymphatic channel (DSLC), and penetrating sentinel lymphatic channel (PSLC) were found in 107 patients. Six lymphatic drainage patterns based on the three types of SLCs were observed in these 107 patients. The proportions of the drainage pattern SSLC, DSLC, PSLC, SSLC+DSLC, SSLC+PSLC, and DSLC+PSLC in the breast were 43%, 0.9%, 15.9%, 33.6%, 3.7% and 2.8%, respectively. The lymphatic drainage pattern in the breast was a significant risk factor for unsuccessful identification of sentinel lymph nodes (*P* **<**0.001) and false-negatives in SLNB (*P* = 0.034) with the subareolar injection technique.

**Conclusions:**

Three kinds of SLCs are the basis of six lymphatic drainage patterns from the breast to the axilla. The type of SLCs is the factor influencing the detection rate and false-negative rate of SLNB. These findings suggest the optimal injection technique of the combination of superficial and deep injection in SLNB procedures. Future clinical studies are needed to confirm our novel findings.

## Introduction

Sentinel lymph nodes (SLNs) can accurately predict axillary lymph nodes status, which is an important prognostic factor in breast cancer [1] and determines the subsequent adjuvant treatment [2]. As a minimally invasive approach, sentinel lymph node biopsy (SLNB), which can reduce postoperative morbidity compared with axillary lymph node dissection (ALND) [3]–[5], has become a standard surgical technique in the management of early invasive breast cancer patients with clinically negative lymph nodes [6], [7].

However, the procedure of SLNB still seems like a “black box”. The tracer is injected into a superficial or deep site and SLNs are identified in the axilla. However, the drainage pattern of the lymphatic system in the breast is unclear. As reviewed by Suami et al, Sappey investigated breast lymphatic drainage with an adult cadaver in 1874 [8], [9]. He observed that the lymphatic's of the breast collected in a subareolar plexus and then drained towards the axilla through lymph collection vessels 8,9. The superficial lymph collection vessel between the areola and SLNs was first defined as the sentinel lymphatic channel (SLC) by Kern et al [10]–[12], and was named the superficial sentinel lymphatic channel (SSLC) by us in this study. Sappey's description of breast lymphatic drainage was universally accepted for nearly 100 years. However, in 1959 Turner-Warwick suggested that the importance of the subareolar plexus was overemphasized because Sappey had mistaken the mammary duct for a lymphatic vessel [8], [13]. He found that the breast drained directly from the tumor to the axilla, which we have named as the deep sentinel lymphatic channel (DSLC). Therefore, the exact route of breast lymphatic drainage to the axilla continues to be debated, although recently Suami et al provided more knowledge on the lymphatic anatomy of the breast [8]. However, like those of Sappey, Suami's findings were based on the anatomy of the normal cancer-free breast in 14 adult cadavers. The controversy regarding lymphatic drainage in the cancerous remains unresolved. To date, no investigators have described the lymphatic anatomy of the cancerous breast using anatomical techniques.

Different lymphatic drainage patterns may help to explain some important unresolved clinical problems, including different detection rates in different studies, and high false-negative rates. Some studies suggest that different detection rates in the SLNB procedure may be caused by different injection sites of tracer [14]–[18]. In addition, false-negative rates of about 10% were reported in multicenter randomized controlled trials [1], [19], [20]. Even these experienced surgeons failed to achieve an acceptable false-negative rate, which is 5% or less according to the 2005 guidelines of the American Society of Clinical Oncology [21]. Suami et al suggested that anatomical studies may help explain the percentages of false-negative SLNBs and identify an appropriate injection site for SLN detection [8]. Therefore, it is important to know more about the drainage patterns of the breast, and even open the “black box” by anatomical studies.

Knowledge of the exact SLCs will provide more underlying information about lymphatic drainage patterns of the cancerous breast. In the present study, we aimed to investigate the exact SLCs in the breast. We dissected 110 specimens of modified radical mastectomy. Interestingly, we first found that both SSLC and DSLC can exist in a single specimen. In addition, a penetrating sentinel lymphatic channel (PSLC) was first observed by us. Therefore, six lymphatic drainage patterns were found based on the three types of SLCs in our patients. These findings may determine whether the type of SLCs has an impact on the detection rate and false-negative rate of SLNB. The results are reported here.

## Patients and Methods

This study was approved by the ethics committee of The First Affiliated Hospital with Nanjing Medical University. All patients provided written informed consent. This study was also in compliance with the Helsinki Declaration. 110 patients with clinically node-negative and stage I or II unifocal breast cancer were included in this study from April 2011 to March 2012. The patients with non-palpable tumors, preoperative chemotherapy, previous axillary node dissection, or excisional biopsy in the upper outer quadrant were excluded. All patients underwent a mimic SLNB procedure, followed by modified radical mastectomy.

### Mimic SLNB procedure

A mimic SLNB procedure in which the blue SLNs were recorded but not removed was performed, followed by modified radical mastectomy. A transverse line marking the SLNB incision was drawn in the axilla about 2 cm below the axillary hair-bearing area. After induction of general anesthesia, 2 mL of methylene blue dye was injected in the subareolar area 16,22 and the injection site was massaged for 5 min. Then, the subcutaneous tissue beneath the marked line was cut open through the transverse skin incision of the modified radical mastectomy. The blue lymphatic channels were inspected, and blue lymph nodes were detected following the route of the blue lymphatic channels. Partially or completely blue-stained lymph nodes, terminations of SLCs, were considered as SLNs. Both SLCs and SLNs were carefully recorded but not excised in the mimic SLNB procedure. Subsequently, modified radical mastectomy with a level II axillary clearance was performed.

### Dissection of all blue lymphatic channels and lymph nodes postoperatively

Methylene blue dye (2 mL) was injected into a single area of the peritumoral parenchyma approaching the axilla immediately after removal of the modified radical mastectomy specimen. If the tumor had been removed by a previous excisional biopsy, the methylene blue dye was injected into a single area of the parenchyma around the biopsy cavity approaching the axilla. The specimen was massaged for 5 min and the location of all blue lymphatic channels and lymph nodes was recorded.

Four surgeons with more than 10 years of experience in breast surgery performed all of the procedures. The mimic SLNB procedure and modified radical mastectomy were performed by two of these four surgeons (X.Z. and X.L.). The other two surgeons examined the specimens for all SLCs and SLNs (M.W. and Yingchun Zhao).

### Evaluation of the mimic SLNB

The number of SLNs recorded introperatively was counted in the mimic SLNB procedure. In this study, the mimic SLNB technique was evaluated by the following parameters: the detection rate was calculated as the number of patients who underwent a successful mimic SLNB divided by the number of patients in whom a mimic SLNB was attempted, the false-negative rate was defined as the number of false-negative patients divided by the sum of the false-negative and true-positive patients, sensitivity was calculated as the number of true-positive patients divided by the sum of true-positive and false-negative patients, accuracy was calculated as the sum of true-positive and true-negative patients divided by the number of patients with successful mimic SLNB.

### Pathological examination

All blue lymph nodes were removed and sent to the pathology laboratory immediately after their anatomic location in the breast was recorded. Each SLN was examined by frozen section and hematoxylin and eosin staining. If any suspicious cells were noted, immunohistochemical (IHC) staining for cytokeratin was used for confirmation of metastasis.

### Statistical analysis

Percentile, median, and range were analyzed for continuous variables. Fisher's exact test and nonparametric rank test were used for univariate analyses. Due to less absolute number, multivariate analyses were not applied for evaluating independent factors that may affect the detection rate and false-negative rate of SLNB. All *P* values were two tailed, and *P*<0.05 was considered statistically significant. All data were analyzed by the software STATA version 11.0 (Stata Corporation, College Station, TX, USA).

## Results

Basic characteristics of the patients are shown in Table 1. The median age of the patients was 56 years (range, 32 to 88 y). No allergic or anaphylactic reactions occurred after the injection of methylene blue dye. All 110 patients underwent modified radical mastectomy after mimic SLNB.

**Table 1:** Patient demographics and primary tumor characteristics.

Characteristic	Number (%)
Age (y)	
Median (range)	56 (32-88)
≤50	39/110 (35.5%)
>50	71/110 (64.5%)
Tumor localization in breast (region)	
Upper outer	49/110 (44.5%)
Upper inner	21/110 (19.1%)
Lower outer	22/110 (20%)
Lower inner	10/110 (9.1%)
Central	8/110 (7.3%)
Tumor size (region)	
≤2 cm	56/110 (50.9%)
>2 cm	48/110 (43.6%)
NA	6/110 (5.5%)
Axillary nodal status (pN)	
Negative	70/110 (63.6%)
Positive	40/110 (36.4%)
Pathology	
Invasive ductal carcinoma	94/110 (85.5%)
Invasive lobular carcinoma	4/110 (3.6%)
Other	8/110 (7.3%)
NA	4/110 (3.6%)
Tumor grade	
1-well differentiated	15/110 (13.6%)
2-moderately differentiated	47/110 (42.7%)
3-poorly differentiated	44/110 (40%)
NA	13/110 (11.8%)

NA:Not available.

With subareolar injection of methylene blue dye, SLNs were successfully identified in 100 patients (100/110, 90.9%). The mean number of SLNs per patient was 1.4 (range, 1 to 3). Of the 100 patients with identifiable SLNs, 37 patients (37%) were node-positive and 5 were false-negative. The false-negative rate was 13.5% (5/37), resulting in a sensitivity of 86.5% (32/37) and accuracy of 95% (95/100).

### Type of sentinel lymphatic channels

**Figure 1:**
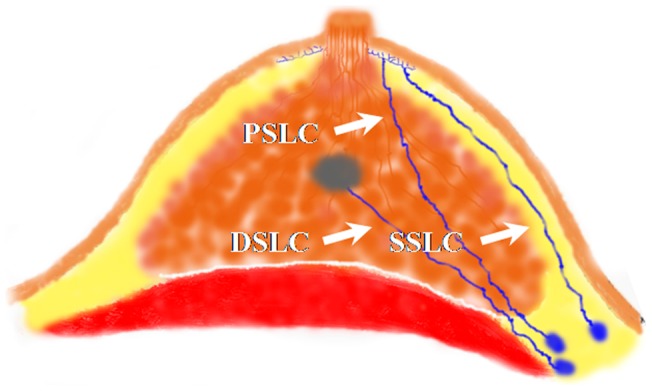
Drawings illustrate three types of SLCs found in the breast from the areolar region or the tumor to the axillary SLNs. However, these three types of SLCs were not found in a single specimen.

**Figure 2:**
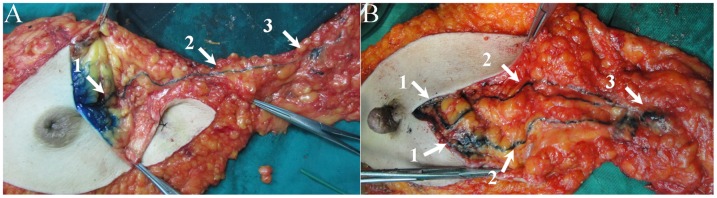
Representative blue SSLCs observed after single subareolar injection of methylene blue dye intraoperatively, showing that pathways of lymph flow arise from the areolar region, pass within the subcutaneous fatty tissue, and terminate at the SLNs in the axilla. A: One blue SSLC from the areolar region to a single blue SLN. B: Two separate SSLCs from the areolar region, through diverging pathways, to two separate but adjacent SLNs. (1. injection site; 2. blue SSLC; 3.SLN).

**Figure 3:**
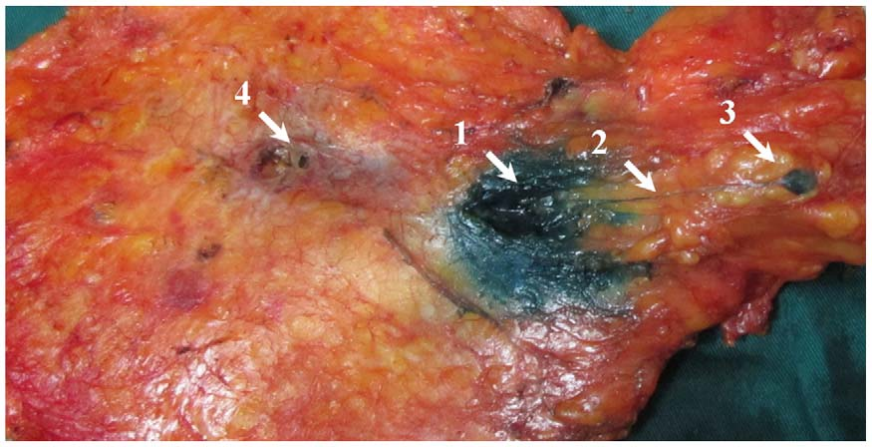
Representative blue DSLCs obtained after single peritumor injection of methylene blue dye postoperatively (back side of the breast), revealing that pathways of lymph flow arise from the peritumor parenchyma, pass through the retromammary tissue above pectoralis deep fascia, and terminate at the SLNs in the axilla. (1. injection site; 2. blue DSLC; 3.SLN; 4.tumor).

**Figure 4:**
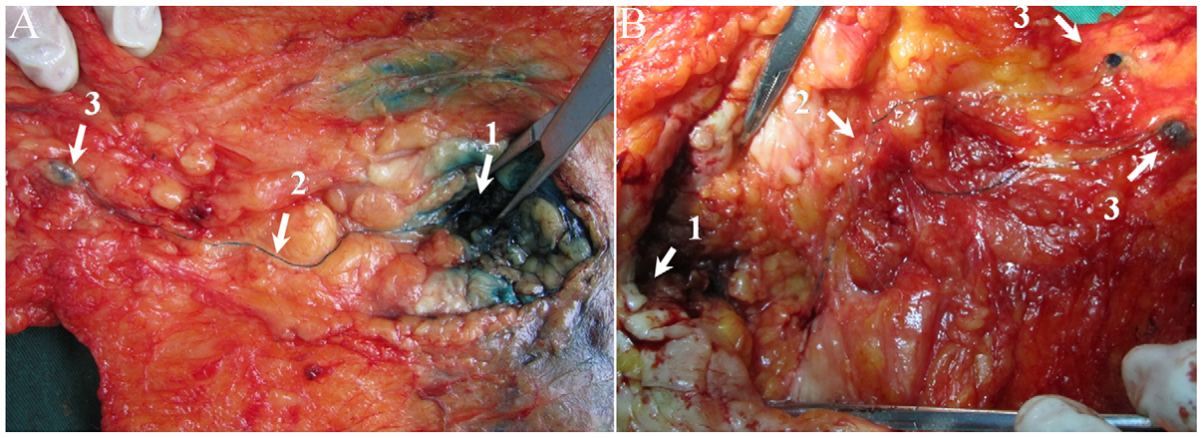
Representative blue PSLCs obtained after single subareolar injection of methylene blue dye intraoperatively (back side of the breast), showing that pathways of lymph flow arise from the areolar region, pass through the breast tissue, and terminate at the SLNs in the axilla. A: A blue PSLC from the areolar region, penetrating the breast tissue, to a single blue axillary SLN. B: A PSLC from the areolar region, passing through the breast tissue, diverging in retromammary tissue, and reaching two separate SLNs. (1. injection site; 2. blue PSLC; 3.SLN).

Lymphatic capillaries of the breast are collected by SLCs, which terminate at axillary SLNs. Three types of SLCs were found in this study (Figure 1), including SSLC, DSLC and PSLC. SSLCs arose from the areolar region, ran upwards in the subcutaneous fatty tissues above the breast parenchyma, and terminated at the axillary SLNs, which lay in a superficial layer of subcutaneous fatty tissue (Figure 2). DSLCs derived from the peritumor parenchyma, ran within the parenchymal tissues of the breast, and reached the axillary SLNs, which lay in a deep layer of subcutaneous fatty tissue and were adjacent to the pectoralis fascia (Figure 3). PSLCs originated from the areolar region, passed through the subcutaneous fatty tissue and parenchyma of the breast, and terminated at the axillary SLNs, which lay in a deep layer of subcutaneous fatty tissue and were adjacent to the pectoralis fascia (Figure 4). Three kinds of SLCs did not interconnect with each other, and remained approximately uniform in diameter until they reached the SLNs (Figure 2, 3, 4). Subareolar injection and the deep injection stained different SLNs respectively.

In addition, blue lymphatic channels leading to the internal mammary nodes were found in three patients during the procedure. This finding was not in accordance with the aim of this study, so the data were not analyzed.

### Type of lymphatic drainage patterns from the breast to the axilla

After methylene blue dye was injected postoperatively, blue lymphatic channels were still not found in three cases. Six kinds of lymphatic drainage patterns from the breast to the axilla (level II) were found in the remaining 107 specimens. Only SSLCs were observed in 46 cases (43%, 46/107) in the breast, and were the anatomical basis of the majority of breast lymphatic drainage patterns. Both SSLCs and DSLCs were observed in 36 cases (33.6%, 36/107) in the breast. Of 24 cases with PSLCs, 17 cases (15.9%, 17/107) had only PSLCs, 4 cases (3.7%, 4/107) had both PSLCs and SSLCs, and 3 cases (2.8%, 3/107) had both PSLCs and DSLCs. The remaining case (0.9%, 1/107) had only DSLC in the breast.

### Unsuccessful detection procedures

Of these 110 patients, 10 experienced unsuccessful SLN detection. Of the 10 specimens removed from these 10 patients with unsuccessful detection, blue lymphatic channels still were not found in 3 specimens on postoperative dissection. Of the remaining 7 specimens, a blue DSLC was found postoperatively in one case, a short blue SSLC was dissected postoperatively in one case, only PSLC was found in 4 cases, and both PSLC and DSLC were found in one case.

The unsuccessful detection rate in patients with the PSLCs was 20.8%, compared with 2.1% in those with the SSLCs (Table 2). The type of SLCs was a significant factor associated with unsuccessful detection of SLNB (*P*<0.001). Patient age, tumor location, tumor size, pathology, and tumor grade did not significantly influence the detection rate of SLNs intraoperatively (*P*>0.05).

**Table 2:** Factors associated with unsuccessful mimic SLNB and false-negative sentinel node biopsy.

Variables	Unsuccessful SLNB (n)	*P* value	False-negative SLNB (n)	*P* value
Patient number	10/110 (9.1%)		5/37 (13.5%)	
Age (years)				
≤50	2/39 (5.1%)	0.490	0/13 (0%)	0.140
>50	8/71 (11.3%)		5/24 (20.8%)	
Tumor location				
Upper outer quadrant	6/48 (12.5%)	0.658	2/11 (18.2%)	1.00
Lower outer quadrant	2/22 (9.1%)		1/7 (14.3%)	
Upper inner quadrant	1/23 (4.4%)		2/12 (16.7%)	
Lower inner quadrant	0/10 (0%)		0/4 (0%)	
Central	1/7 (14.3%)		0/3 (0%)	
Tumor size				
≤2 cm	5/56 (8.9%)	0.797	3/18 (16.7%)	1.00
>2 cm	5/48 (10.4%)		2/17 (11.8%)	
Pathology				
Invasive ductal	9/94 (9.6%)	0.716	5/32 (15.6%)	1.00
Invasive lobular	0/4 (0%)		0/3 (0%)	
Other	1/8 (12.5%)			
Tumor grade				
1-well differentiated	3/15 (20%)	0.347	0/1 (0%)	1.00
2-moderately differentiated	4/47 (8.5%)		3/19 (15.8%)	
3-poorly differentiated	3/44 (6.8%)		2/16 (12.5%)	
Type of SLC				
SSLC	1/46 (2.2%)	**<0.001**	0/15 (0%)	**0.034**
PSLC	5/24 (20.8%)		1/10 (10%)	
SSLC+DSLC	0/36 (0%)		4/12 (33.3%)	
DSLC	1/1 (100%)		/	

### False-negative rate of SLNB

Of the 37 patients with positive lymph nodes in mimic SLNB, 15 patients had only SSLC, 10 had PSLC, and 12 had both SSLC and DSLC (Table 2). Of the 5 false-negative cases, both SSLC and DSLC were found in 4 cases, and PSLC was found in the remaining case. The false-negative rate in patients who had both SSLCs and DSLCs was 33.3%, compared with 10% in patients with PSLCs, and 0% in patients with only SSLC (*P* = 0.034). However, patient age, tumor location, tumor size, pathology, and tumor grade did not significantly affect the false-negative rate in SLNB procedures (*P*>0.05).

## Discussion

SLNB is a useful procedure that minimizes the morbidities associated with ALND. However, the procedure of SLNB still seems like a “black box”. In this study, the “black box” of SLNB was opened a little by our finding of three types of SLCs – SSLC, DSLC and PSLC. Six drainage patterns based on the three types of SLCs also were observed. Furthermore, the type of SLCs was associated with the detection rate and false-negative rate of SLNB.

The concept of SLCs was first suggested by Kern et al., who reported that the SLCs interconnected the areola and axillary SLNs [10]–[12]. The finding of SSLCs in this study corresponds to Kern et al. 's concept of SLCs. SSLCs became the theoretical basis for the subareolar injection of tracer in the lymphatic mapping procedure. Both SSLCs and a subareolar lymphatic plexus also were the anatomical basis of Sappey's theory [8], [9].

However, the existence of additional collecting lymphatic vessels may have been neglected by Sappey and Kern [8]–[12]. The DSLC is another type of SLC. DSLCs in the present study may be consistent with the findings of Turner-Warwick and Tanis PJ [8], [13], [23]. These investigators reported that the lymphatic trunks between the tumor and the axillary SLNs had a direct course in most patients. However, DSLCs in our study did not agree with the deep lymphatic system described by Suami et al. [8], who reported that the deep lymphatic system was beneath the deep fascia. In our study, the DSLCs observed in 40 patients (37.4%) lay in breast parenchyma above deep fascia.

In addition, PSLC – a new lymphatic drainage channel – was found. This type of lymphatic vessel was not reported previously. The PSLC was not in agreement with the perforating lymphatic system demonstrated by Suami et al. [8], who reported that the perforating lymphatics pierced the intercostal fascia, connected to the deep lymphatic system, coursed with the internal mammary artery and vein, and ended in internal mammary nodes. However, in the present study, the PSLC penetrated breast parenchyma and reached the axillary SLNs without piercing the deep fascia.

Our findings provide good support for Kern's concept of SLCs. We defined the SLCs as the collecting lymphatic channels between the areola or the tumor and axillary SLNs. Three types of SLCs were the anatomical basis for six kinds of breast lymphatic drainage patterns, which were found in 107 patients. These three breast SLCs were not interconnecting. Therefore, the breast lymph drainage patterns to axillary SLNs via SLCs were independent. There should be no controversy regarding lymphatic pathways of the mammary gland to the axilla, since different lymphatic drainage patterns are likely to result from differences among patients.

In this study, it was found that the type of SLCs could affect the detection rate of SLNB. However, the results of this study suggest that patient age, tumor location, tumor size, pathology, and tumor grade do not affect the detection rate of SLNB. Of 10 patients with unsuccessful SLNB, blue lymphatic channels were found in 7 specimens on postoperative dissection. Five of the 7 patients in whom detection with blue mapping was unsuccessful had PSLCs. In these five patients, the location of the SLNs connected with PSLC was deep in the subcutaneous fatty tissue, resulting in unsuccessful detection of SLNs. If the surgeons did not know the concept of the PSLC, they might easily have failed to identify the deep SLNs in patients with only PSLC. Additionally, SLNB failed in one patient with only SSLC because the SSLC was short and the SLN connected with the SSLC was far away from the incision.

With superficial (including subareolar, intradermal, and subdermal) injection of methylene blue dye, mapping failure may occur in the patient with only DSLC [24]. In our study, this disturbing phenomenon was exemplified by one patient who had only DSLC. With deep (including peritumoral and intratumoral) injection of methylene blue dye, mapping failure may occur in the patient with SSLC or PSLC or both SSLC and PSLC [24]. When the injection site of tracer is selected, the proportions of different types of SLCs result in different detection rates of SLNB. These possible results are not associated with the surgeon's proficiency or technique. Therefore, we infer that the superficial injection technique will show a higher detection rate of SLNB compared with a deep injection technique in most patients with SSLCs, and the deep injection technique is superior to the superficial injection technique in identifying SLNs in most patients with DSLCs. In most patients with both SSLC and DSLC the detection rates of SLNs with deep or superficial injection techniques are the same.

This is the first report that the type of SLCs is a significant factor in false-negative rates of SLNB. However, patient age, tumor location, tumor size, pathology, and tumor grade did not influence the false-negative rate of SLNB. Of 5 false-negative cases, 4 had both SSLC and DSLC. In those patients in whom methylene blue was injected into the subareolar area, no blue DSLCs were observed introperatively. This phenomenon possibly occurred in 36 patients with both SSLC and DSLC. Except for the 4 false-negative cases, the remaining patients were potential false-negative cases. Since these patients were diagnosed with early-stage breast cancer and most patients were lymph node negative, a false-negative rate of 13.5% was observed. We inferred that a higher false-negative rate might be observed in later-stage breast cancer patients. In several multicenter studies, the high false-negative rates were possibly related to a lack of lymphatic mapping of SSLC because of a deep injection site [19], [20]. With regard to the patients with both SSLC and DSLC, either a superficial or deep injection technique resulted in a high false-negative rate. It is possible that even surgeons with long-time surgical experience could not avoid false-negative findings.

Our study has some limitations. First, only blue dye was used, which may lead to a high missed SLN identification rate. However, blue dye can make the SLCs clearly. Second, deep injection of blue dye was performed postoperatively. Future studies should be undertaken to confirm our finding intraoperatively. Third, the sample size in the present study was small, and multivariate analysis was not performed to adjust for other factors. Fourth, lymphatic capillaries may exist among the SLCs in the breast. However, the existence of lymphatic capillaries cannot be confirmed in our study. Fifth, the lymphatic capillaries between the tumor and subareolar lymphatic plexus could not be demonstrated in our study. Detailed information about the lymphatic system in the breast should be the subject of future investigations. Sixth, since IHC was performed only when suspicious cells were noted, the rate of SLN metastasis may be underestimated in the present study.

In conclusion, our study provides important information about SLNB. Three kinds of SLCs are the basis of six lymphatic drainage patterns from the breast to the axilla. The type of SLCs is the factor influencing the detection rate and false-negative rate of SLNB. The anatomical type of SLCs is the underlying basis for the success of lymphatic mapping. The combination of superfical and deep injection techniques may be optimal for tracer injection in SLNB procedures. Future clinical studies are needed to confirm our novel findings.
